# The Preparation of Green Fluorescence-Emissioned Carbon Dots/Poly(N-Isopropylacrylamide) Temperature-Sensitive Hydrogels and Research on Their Properties

**DOI:** 10.3390/polym11071171

**Published:** 2019-07-11

**Authors:** Dan Zhao, Wenting Ma, Rong Wang, Xinzhou Yang, Jun Li, Ting Qiu, Xincai Xiao

**Affiliations:** 1 School of Pharmaceutical Sciences, South-Central University for Nationalities, Wuhan 430074, China; 2 National Demonstration Center for Experimental Ethnopharmacology Education, South-Central University for Nationalities, Wuhan 430074, China; 3 Information Center, Shanghai Institude of Organic Chemistry, Shanghai 200032, China

**Keywords:** temperature-sensitive hydrogel, green-emissioned carbon dots, preparation of hydrogel, fluorescence ink

## Abstract

Fluorescence/temperature-sensitive hydrogels, thanks to their properties in fluorescence and temperature sensitivity, have shown a promising outlook in the fields of drug delivery, cell imaging, etc., and thus become the focus of present research. This paper reports the preparation of green-fluorescence/temperature-sensitive hydrogels through one-step radical polymerization with green fluorescence-emissioned carbon dots as fluorescence probes and N-isopropylacrylamide as a monomer. UV-vis spectra, fluorescence spectra, and fluorescence microscope imaging have been used to characterize the prepared hydrogel, and to study their optical and temperature-sensitive properties. It was discovered that the emission of prepared hydrogel is excitation wavelength-dependent, pH responding, and excellent temperature-sensitive, as well as having good biocompatibility. The prepared hydrogel can also be applied as fluorescence ink in the fields of anti-counterfeit identification and appraisal.

## 1. Introduction

Thanks to its excellent biocompatibility [1,2], optical stability [1], low toxicity [1,2], and temperature-sensitivity [1,2], fluorescence/temperature-sensitive hydrogels attract increasing research interest as a new multi-functional smart material, exhibiting promising outlook in the fields [1,3,4,5] of drug delivery, chemical sensor, and biological imaging. The hydrogels have a great range of types, and their monomers are mainly N-isopropylacrylamide (NIPAM), N-vinylcaprolactam, acrylamide, etc. [6,7,8,9]. NIPAM is one of the most studied temperature-sensitive monomers due to its excellent biocompatibility, low critical solution temperature (LCST), and it being close to human body temperature. With NIPAM as a raw material, the prepared temperature-sensitive hydrogel is an excellent carrier for drug delivery. The usual embedded fluorescence materials [3,4,10,11,12] in fluorescence/temperature-sensitive hydrogels are fluorescent dyes, nano metal clusters, quantum dots (QDs), etc. QDs have been the focus for researchers in the field of fluorescent nanomaterials because of their unique optical properties. In the early stage, II-VI group QDs were applied in the synthesis of fluorescence/temperature-sensitive hydrogels. In 2016, Cai et al. [13] prepared fluorescence/temperature-sensitive hydrogels by mixing and stirring CdTe QDs and poly(N-isopropylacrylamide) at room temperature and in a certain pH environment. Liras and his team [14] used methacrylic acid group-modified CdSe QDs and poly(diethylene glycol methyl ether methacrylate) as raw materials and prepared temperature sensitive smart hydrogel through free radical polymerization. These types of synthesis methods are simple in operation, and the prepared hydrogel exhibits reversible temperature-dependent fluorescence. Some hydrogels have excellent stability as a carrier and a high drug load capacity. However, the Cd element in cadmium based QDs is potentially threatening human health and the environment [15,16], which limits the application of prepared hydrogel in the fields of biological medicine and food safety.

Carbon dots (CDs) have attracted widespread attention from the researchers thanks to their heavy-metal free, raw material richness, simple synthesis process, excellent optical stability and biocompatibility, and low toxicity [17,18,19,20,21]. Campos et al. [22] used 3-butenoic acid to modify the green-emissioned polyethylene glycol-modified carbon dots (G-PEG-CDs), and introduced the modified CDs into temperature-sensitive poly(N-isopropyl acrylamide) (PNIPAM) hydrogels through free radical polymerization to get fluorescent/temperature-sensitive complex hydrogels. Li and his team [3] used blue-emissioned CDs to copolymerize with NIPAM to get fluorescent/temperature-sensitive hydrogel, and applied it into cell imaging. Kim et al. [12] used blue-emissioned CDs, fluorescein, and PNIPAM as raw materials to acquire core-shell nano-hydrogel through emulsion copolymerization. However, the CDs used in the preparation of fluorescent thermosensitive hydrogels in the early stage were mainly B-CDs. In recent years, more and more long-wavelength CDs have been prepared. Compared with B-CDs, they have wider excitation spectra, and their excitation wavelength is tunable according to the background fluorescence of the organism in order to get high-contrast pictures. Our team has prepared long-wavelength green-emissioned CDs [23] (G-CDs, QYs = 22.2%) with polyethyleneimine, citric acid, and ascorbic acid as raw materials. G-CDs are used as fluorescent probes in the synthesis of fluorescent temperature sensitive hydrogels, which provide new methods and materials for drug delivery and drug carrier candidates.

This paper reports the one-step preparation of CDs/PNIPAM fluorescence/temperature-sensitive hydrogel with G-CDs, acrylic acid (AAc) and NIPAM as raw materials, N,N’-methylenebisacylamide (BIS) as a crosslinking agent, and potassium persulphate (KPS) as the initiator through polymerization reaction (oil bath, 70 °C). The typical properties of prepared CDs/PNIPAM, such as optical properties, temperature sensitivity, and dependence on pH environment, have been studied. The prepared hydrogel has been applied in fluorescence imaging, ink, and anti-counterfeiting identification. This research would provide new material for a wide range of fields like temperature-sensitive sensors, drug carrier system, fluorescent labeling, fluorescent coding, and fluorescent ink.

## 2. Experimental Section

### 2.1. Materials and Apparatus

N-isopropylacrylamide (NIPAM), N,N’-methylenebisacrylamide (BIS), and ethylene imine polymer (PEI_1800_) were obtained from Aladdin Chemistry Co., Ltd. (Shanghai, China); acrylic acid (AAc), potassium persulphate (KPS), L-ascorbic acid (AA), citric acid monohydrate (CA), and dimethyl sulfoxide (DMSO) were purchased from Sinopharm Chemical Reagent Co., Ltd. (Shanghai, China); cyclohexane was obtained from Tianjin Bodi Chemical Co., Ltd. (Tianjin, China); and acetone was purchased from Tianjin Fuyu Fine Chemical Co., Ltd. (Tianjin, China). Tris–HCl buffer solutions with different pH values were prepared by dropwise and added into a concentrated hydrochloric acid solution (0.1 mol·L^−1^) or tris solution (20 mmol·L^−1^) to required pH values. L02 cell was purchased from Tongpai Biological Technology Co., Ltd. (Shanghai, China). All chemicals used were of analytical grade or of the highest purity available. Deionized water was prepared from a Milli-Q-RO4 water purification system (Millipore, Burlington, MA, USA).

UV-Visible absorption spectra were acquired with a Lambda-35 UV-Visible spectrophotometer (PerkinElmer Company, Waltham, MA, USA) to determine the bandgap absorption of CDs/PNIPAM. Fluorescence spectra were recorded on a LS55 spectrofluorometer (PerkinElmer Company, Waltham, MA, USA). Experiments on the fluorescence emission as a function of temperature were conducted, ranging from 25–43 °C. The pH was monitored by a PHSJ-3F pH meter (Shanghai Precision Scientific Instrument Company, Shanghai, China). Zeta-potential measurement was carried out on a Zetasizer nanoseries ZEN3690 (Malvern Instruments Ltd., Malvern, UK). Images of fluorescent ink were lit on a ZF-1 three-use UV analyzer (Shanghai Jinpeng Analytical Instruments Co., Ltd., Shanghai, China). The TEM images were obtained at 310 K magnification with a FEI Tecnai G2 20 s-twin transmission electron microscope (FEI Company, Hillsboro, OR, America). Fluorescence lifetime was measured with an FLS920 fluorescence spectrometer (Edinburgh Instruments Ltd., Edinburgh, UK). Microscope images were acquired with the XD30-RFL microscope (Ningbo Sunny instruments Co., Ltd., Zhejiang, China).

### 2.2. Synthesis of G-CDs

G-CDs used in experiments were synthesized by our laboratory [23]. A measure of 0.15 g of AA, 0.18 g of PEI_1800_, and 0.8 g of CA were dissolved in 10 mL of distilled water, and the mixture was stirred at room temperature for 5 min. The solution was then transferred to an autoclave and further reacted at 150 °C for 1 h.

### 2.3. Synthesis of PNIPAM Microgels

Both NIPAM and BIS samples must be recrystallized and purified before use. A measure of 0.124 g of NIPAM, 0.0062 g of BIS, and 21 μL of AAc were dissolved in 13.04 mL of deionized water within the flask equipped with a mechanical stirrer and purged with nitrogen for 30 min at 70 °C. The polymerization was initiated by the addition of 0.02 g of KPS dissolved in 0.5 mL of water. The solution was heated to 70 °C with nitrogen for 30 min. After the flask was sealed, the reaction was continued for another 4 h before being cooled down naturally. After cooling, the microgels were purified by ultrafiltration at 8000 rpm for 10 min, and dispersed in deionized water. The PNIPAM hydrogels were obtained.

### 2.4. Synthesis of CDs/PNIPAM Microgels

A measure of 0.124 g of NIPAM, 0.0062 g of BIS, 21 μL of AAc, and 1 mL of G-CDs were dissolved in 13.04 mL of deionized water within the flask equipped with a mechanical stirrer and purged with nitrogen for 30 min at 70 °C. The polymerization was initiated by the addition of 0.02 g of KPS dissolved in 0.5 mL of water. The solution was heated to 70 °C with nitrogen for 30 min. After the flask was sealed, the reaction was continued for another 4 h before being cooled down naturally. After cooling, the microgels were purified by ultrafiltration at 8000 rpm for 10 min, and dispersed in deionized water. The CDs/PNIPAM hydrogels were obtained.

### 2.5. Cytotoxicity Assay

To analyze the in vitro cytotoxicity of CDs/PNIPAM, L02 cells were seeded in 96-well plates with a density of about 1 × 10^4^ cells per well for 24 h. The sample of CDs/PNIPAM was dissolved in deionized water, and DMEM medium was added to prepare different concentrations. Different concentrations of diluted samples were added to each well (DMSO concentration was less than 1‰). The cells were incubated for a further 24 h. After the supernatant was discarded, 100 μL of MTT solution was added to each well, and the mixture was placed in an incubator for a further 30 min. The supernatant was discarded and 150 μL of DMSO was added to each well. They were shaken in the dark for 10 min. The microplate reader detected the optical density (OD) at 562 nm.

(1) $$\mathrm{Cell}\;\mathrm{survival}\left(\%\right)=\;\frac{\mathrm{OD}\;\mathrm{value}\;\mathrm{of}\;\mathrm{experimental}\;\mathrm{group}\;-\;\mathrm{OD}\;\mathrm{value}\;\mathrm{of}\;\mathrm{blank}\;\mathrm{control}\;\mathrm{group}}{\mathrm{OD}\;\mathrm{value}\;\mathrm{of}\;\mathrm{negative}\;\mathrm{control}\;\mathrm{group}\;-\;\mathrm{OD}\;\mathrm{value}\;\mathrm{of}\;\mathrm{blank}\;\mathrm{control}\;\mathrm{group}}\times 100\%$$

## 3. Results and Discussion

### 3.1. The Preparation of CDs/PNIPAM Complex Hydrogel

**Figure 1:**
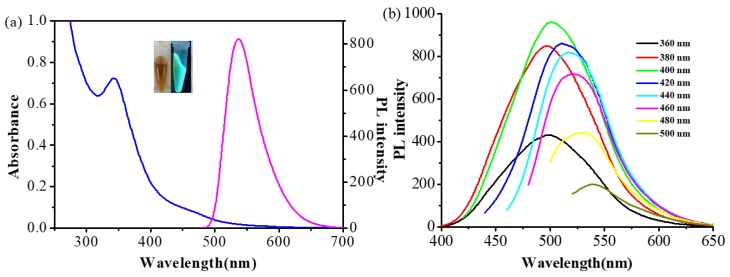
(**a**) UV-Vis spectrum and fluorescence emission spectrum of G-CDs; (**b**) Fluorescence emission spectra of G-CDs solution with different excitation wavelengths.

The fluorescence probe used in this research is the G-CDs with PEI as the carbon source, and their UV-Vis and fluorescence spectra are shown in Figure 1. The UV-Vis spectrum shows a sharp absorption peak at 350 nm. When the excitation wavelength is 480 nm, the emission peak of G-CDs (0.13 mg/mL) is 537 nm (Figure 1a). When excitation wavelength changes from 360 to 500 nm, the maximum emission peak of G-CDs redshifts, and its fluorescence intensity increases then drops. The optimal excitation wavelength is 400 nm when fluorescence intensity reaches maximum, which shows the excitation wavelength-dependent property of G-CDs (Figure 1b).

**Figure 2:**
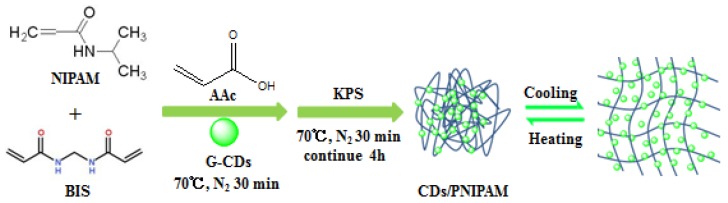
Synthesis and phase transition diagram of CDs/PNIPAM.

Figure 2 is a schematic diagram of the synthesis and phase transition of CDs/PNIPAM. With G-CDs as a fluorescence probe, AAc and NIPAM as raw materials, BIS as crosslinking agent, and KPS as initiator, the CDs/PNIPAM fluorescence/temperature-sensitive hydrogel was acquired through polymerization reaction (oil bath, 70 °C, Figure 2). The prepared product was purified through ultrafiltration. When the external temperature is lower than the LCST, this compsite hydrogel swells with fluorescence increase; when the external temperature is higher than the LCST, the hydrogel shrinks with fluorescence decrease.

**Figure 3:**
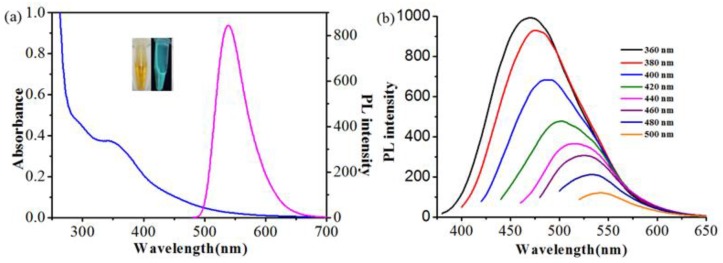
(**a**) UV-Vis spectrum and fluorescence emission spectrum of CDs/PNIPAM; (**b**) Fluorescence emission spectra of CDs/PNIPAM solution with different excitation wavelengths. Inset: Fluorescence image of the hydrogel under daylight (left) and 365 nm UV light (right).

As shown in Figure 3a, CDs/PNIPAM (0.92 mg/mL) exhibits UV absorption peak at 350 nm. Though the UV absorption peak of this composite hydrogel is identical to that of the naked G-CDs, the shape of the absorption peak of this hydrogel (pointed peak) is different from that of the naked G-CDs (curved peak) because of the change of micro-environment of G-CDs when embedded in hydrogel. Since PNIPAM hydrogel itself does not have a UV absorption peak and fluorescence (Figure S1), G-CDs can thus be proven to be embedded into the hydrogel. When the excitation wavelength is 480 nm, the emission wavelength is 537 nm (Figure 3a). This hydrogel solution emits bright green fluorescence (Inset). The position of the fluorescent emission peak of the composite hydrogel does not change significantly compared to the naked G-CDs. Comparing the fluorescence spectra of CDs/PNIPAM hydrogels with G-CDs at different excitation wavelengths, it can be found that the trends of fluorescence intensity changes are significantly different. The optimal excitation wavelength of the naked G-CDs changes from 400 to 360 nm, and a blue shift occurs. The fluorescence spectra also show that CDs/PNIPAM exhibit classic excitation-wavelength-dependent photoluminescence characteristics.

### 3.2. Optical Properties

**Figure 4:**
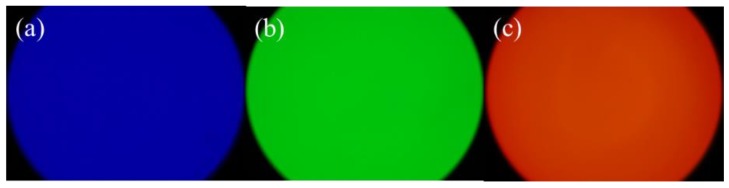
Fluorescence images of CDs/PNIPAM irradiated by different light sources: (**a**) ultraviolet light, (**b**) blue light, and (**c**) green light.

The fluorescence microscopy imaging of prepared CDs/PNIPAM hydrogel under different excitation wavelength was investigated. As shown in Figure 4, the hydrogel exhibits blue fluorescence under the irradiation of UV light, green fluorescence under blue light, and red fluorescence under green light, showing that the doping of CDs endows the fluorescence property of prepared CDs/PNIPAM hydrogel. The ability to show multiple-colored fluorescence under the irradiation of different excitation wavelengths would make it possible to apply it to the field of multiple-colored fluorescence imaging. Since the penetration ability through the organism differs for different wavelength light, the penetration ability increases with wavelength [24], with higher resolution and lower background interference, and more beneficial to biological imaging. Therefore, this property would ensure its promising outlook in fields like biological and cell imaging.

The fluorescence lifetime was then tested according to the double exponential function of nonlinear least squares analysis to fit the fluorescence decaying curve of prepared CDs/PNIPAM hydrogel:

I(t) = A_1_·exp(−t/τ_1_) + A_2_·exp(−t/τ_2_) (2)

where τ_1_ and τ_2_ are decay time constant, and A_1_ and A_2_ are the ratios of time resolved decay lifetime that τ_1_ and τ_2_ take. The average fluorescence lifetime of CDs/PNIPAM (τ_avg_) hydrogel was calculated according to the equation:

τ_avg_ = (A_1_·τ_1_^2^ + A_2_·τ_2_^2^)/(A_1_·τ_1_ + A_2_·τ_2_) (3)

**Figure 5:**
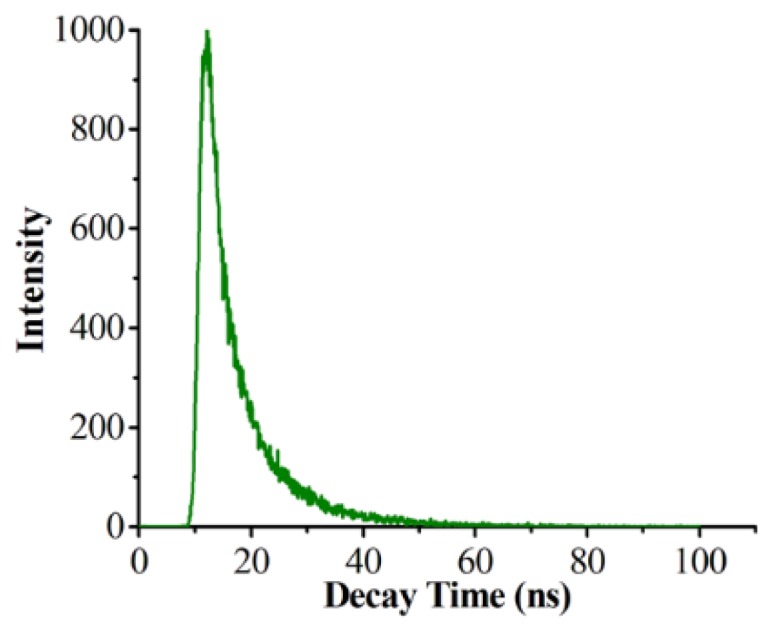
Fluorescence decay curves of the CDs/PNIPAM.

According to the fitting result in Figure 5, there is short lifetime (τ_1_) and a long lifetime (τ_2_) for CDs/PNIPAM hydrogel. The double exponential behavior of fluorescence lifetime shows the existence of two different emission positions (fast decay component), corresponding to the recombination of surface state and molecular state. Compared with molecular state, the emissions originating from surface state usually exhibit shorter recombination lifetime. Therefore, it can be inferred that τ_1_ originates from the surface state of CDs of the surface, while τ_2_ is from the molecular state, and the average lifetime is calculated to be 8.21 ns.

### 3.3. The Analysis of TEM, Zeta Potential and FTIR Spectroscopy

**Figure 6:**
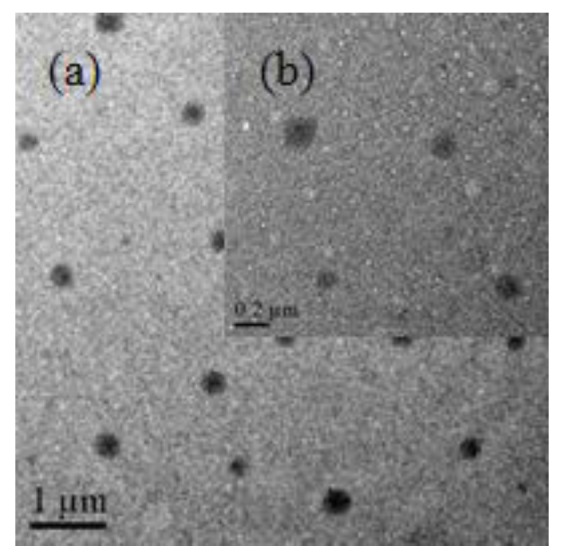
The TEM images of CDs/PNIPAM. (**a**) Overall view; (**b**) Enlarged view.

The TEM image of CDs/PNIPAM hydrogel is shown in Figure 6. It can be shown that CDs/PNIPAM hydrogels are in sphere shape with uniform distribution. The average particle size is about 294 nm. The deep colored region in the sphere shows that G-CDs might be embedded inside as well as on the surface of the hydrogel.

The surface potential of nano-particles can be measured and analyzed by zeta potential. The zeta potentials of G-CDs, PNIPAM hydrogel, and CDs/PNIPAM were measured separately. As can be seen from Table 1, the Zeta potential of G-CDs is +11.4 mV. The rich amino groups of the raw material PEI endow large amounts of NH_4_^+^ on the surface of prepared G-CDs, making it positively-charged in this environment. The zeta potential of PNIPAM hydrogel is −14.4 mV because of the carboxyl group in the raw materials of hydrogel (NIPAM, AAc). The zeta potential gets an obvious decrease (−3.36 mV) in CDs/PNIPAM hydrogel because the positive-charged G-CDs were embedded into the hydrogel, leading to the decrease of absolute potential. 

**Figure 7:**
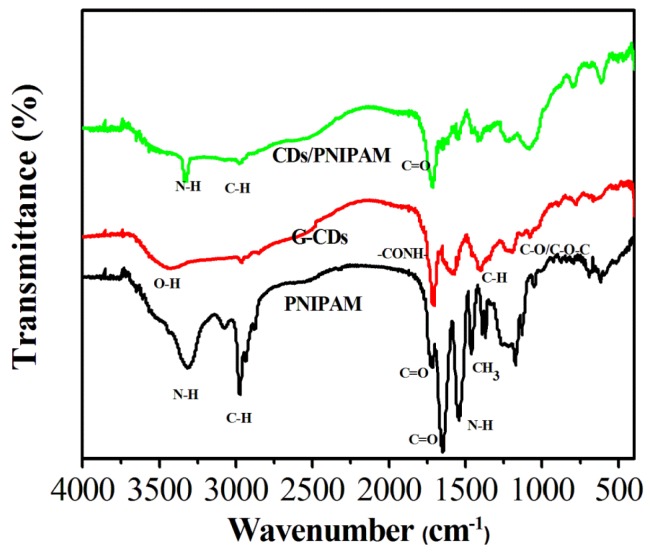
FTIR spectra of CDs/PNIPAM, G-CDs, and PNIPAM.

FTIR was used to characterize the functional groups on the surface of CDs/PNIPAM hydrogel. As shown in Figure 7, in the infrared spectrum of CDs/PNIPAM hydrogel, the absorption peak at 3330 cm^−1^ originates from the stretching vibration of N–H, and the peak at 2980 cm^−1^ originates from the stretching vibration of C–H; the peak at 1720 cm^−1^ is from the —CONH-group, and the peaks at 1640 and 1560 cm^−1^ are the stretching vibration of C=O and the in-plane bending vibration of N–H. The characteristic peaks of PNIPAM hydrogel are basically in accordance with those of CDs/PNIPAM hydrogel. The absorption peaks at 1420 and 1220 cm^−1^ originate from C–H and C–O/C–O–C groups from G-CDs. This shows that G-CDs do not only exist on the surface of prepared CDs/PNIPAM hydrogel, but also are embedded inside the hydrogel. The CDs/PNIPAM hydrogel is successfully prepared.

### 3.4. The Temperature-Sensitive Performance

**Figure 8:**
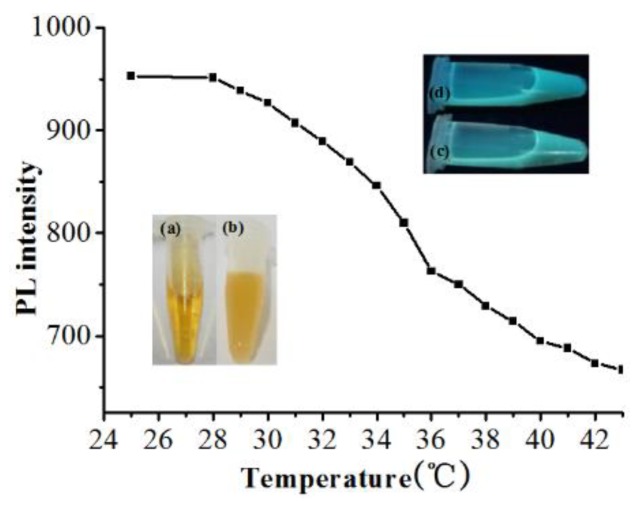
The diagram of fluorescence intensity of CDs/PNIPAM with different temperatures. (**a**) 25 °C under daylight; (**b**) 40 °C under daylight; (**c**) 25 °C under ultraviolet; (**d**) 40 °C under ultraviolet.

Since temperature sets an obvious impact upon the fluorescence properties of prepared CDs/PNIPAM hydrogel, the temperature-sensitive performance of the hydrogel was investigated. The temperature of CDs/PNIPAM hydrogels was controlled in the range of 25–43 °C through water-bathing to study the fluorescence intensity change with temperature (λ_ex_ = 480 nm). As shown in Figure 8, the fluorescence of the hydrogel decreases with the rise of temperature, and it drops sharply when temperature is higher than the LCST. The original yellow clear solution would become turbid (Figure 8b) with fluorescence decrease (Figure 8d and Figure S2). The simple PNIPAM particle also exhibits similar properties (Figure S3). This may be the result of the shrinking state caused by the increased hydrophobic effect of the hydrogel under high temperature, leading to high concentration of CDs in certain areas of hydrogel, and the decrease of overall fluorescence intensity of hydrogel. Moreover, the shrinking state of the hydrogel would also greatly increase its absorption, refection, and scattering of light, turning it a strong light scattering center [25] and thus reducing its overall fluorescence. This temperature sensitive phenomenon would be useful to develop temperature-sensitive sensors, temperature-sensitive equipment, and temperature control valves.

**Figure 9:**
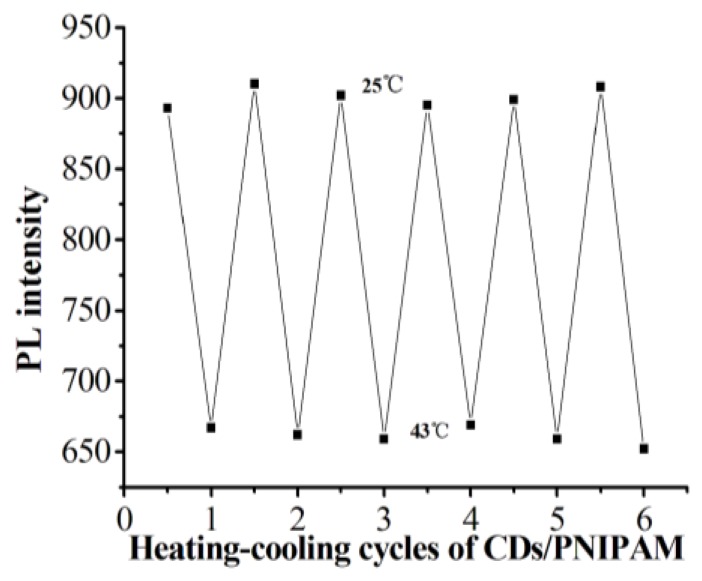
The change in fluorescence intensities of the CDs/PNIPAM aqueous solution during heating/cooling cycles between 25 and 43 °C.

To investigate the reversibility and repeatability of the temperature-sensitive fluorescence of CDs/PNIPAM hydrogel, the hydrogel was heated to 43 °C and then cooled down to 25 °C six times, and the fluorescence spectra were measured. As shown in Figure 9, with the increase of temperature, the fluorescence of the CDs/PNIPAM shows the “off” state, while it is “on” when the temperature drops. This temperature-sensitive property exhibits excellent reversibility. Moreover, after many repeats of the temperature rise-drop process, the fluorescence intensity of CDs/PNIPAM hydrogel still shows obvious dependence on temperature, with good repeatability, proving the reusability of prepared hydrogels.

### 3.5. pH-Sensitive Performance

Before studying the fluorescence intensity of CDs/PNIPAM hydrogel in different pH environments, the stability of G-CDs was first examined. As shown in Figure S4, the fluorescence stays stable when pH ranges from 3 to 11. Then, with the excitation wavelength at 480 nm, the fluorescence intensities of CDs/PNIPAM hydrogel in different pH environments was measured to study it’s changing pattern with pH value.

**Figure 10:**
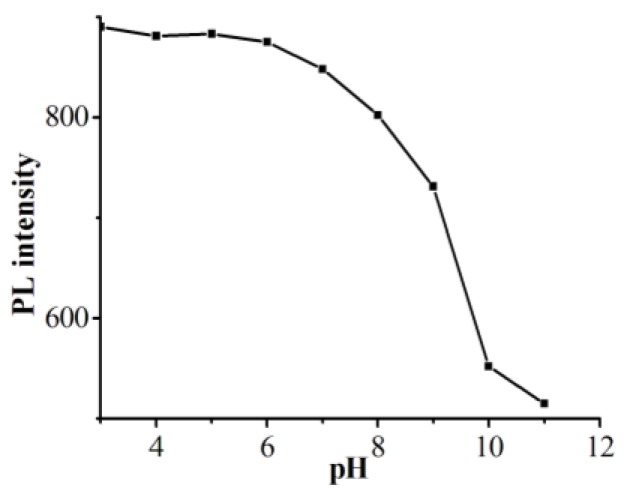
Fluorescence intensity of CDs/PNIPAM at different pH conditions.

As shown in Figure 10, the fluorescence intensity is relatively stable when pH is in the range of 3–5, while the intensity drops with further increase of pH value, and it exhibits a sharp drop at pH 9–10, showing the hydrogel is stale in an acidic environment and sensitive in an alkalescent one. When the pH environment changes, the turbidity of the PNIPAM particle did not change significantly (Figure S5). This phenomenon may be the result of bonding interaction changes on the surface of CDs. The increase of pH would induce the change of the electrolytic environment of hydrogel-capped CDs, leading to changes in the surface structure and the state of G-CDs, and thus the change of fluorescent properties. Moreover, this fluorescence change might also relate to the swelling and contracting of hydrogels in different pH environments [26].

### 3.6. The Application of CDs/PNIPAM

#### 3.6.1. Biocompatibility of CDs/PNIPAM

**Figure 11:**
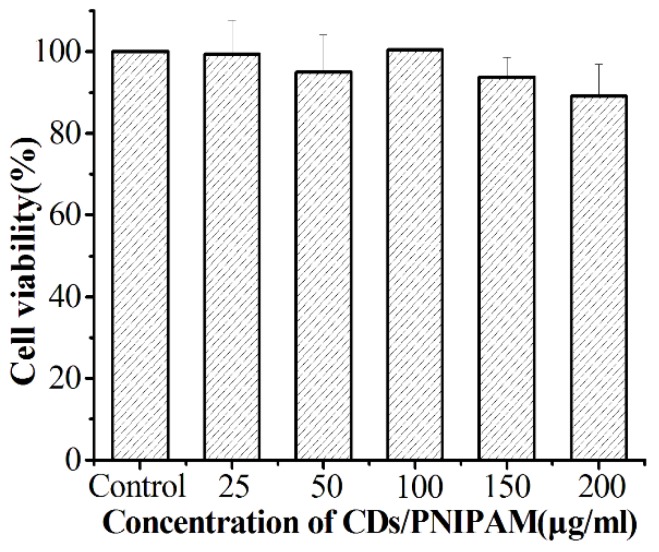
Cell viability of CDs/PNIPAM incubated with L02 cell for 24 h.

Good biocompatibility is a prerequisite for the application of CDs/PNIPAM hydrogels to drug delivery. The biocompatibility of CDs/PNIPAM hydrogels was examined by an MTT assay. As shown in Figure 11, the toxicity test of CDs/PNIPAM hydrogels on L02 normal cells can be seen; when the concentration of the hydrogel increased from 25 to 200 μg/mL, the cell survival rate was always above 89%. This indicates that the CDs/PNIPAM hydrogel has less effect on the growth of L02 normal cells. The hydrogel has good biocompatibility. The good biocompatibility of this composite hydrogel ensures its promising potential as drug carrier material in drug delivery and other biomedical fields.

#### 3.6.2. Fluorescent Ink

**Figure 12:**
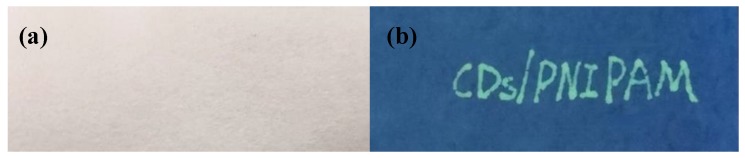
Written “CDs/PNIPAM” on filter paper by CDs/PNIPAM used as the fluorescent ink. (**a**) Daylight lamp; (**b**) 365 nm ultraviolet lamp.

The CDs/PNIPAM prepared herein could be applied to fluorescent inks. The hydrogel ink was written on filter paper and photographed under a 365 nm UV lamp. As shown in Figure 12, under room temperature, 0.92 mg/mL pH = 5 CDs/PNIPAM hydrogels fluorescent ink was written on filter paper and photographed under 365 nm UV light. The English letters “CDs/PNIPAM” showed green fluorescence under the ultraviolet light and the words were clear and bright. It had no trace under the daylight lamp. Due to this property, the fluorescent/thermosensitive hydrogel which was easy to obtain, non-toxic, environmentally friendly, and had great development potential in identification and anti-counterfeiting.

## 4. Conclusions

This paper reports the synthesis of CDs/PNIPAM hydrogel with excellent fluorescence property and temperature-sensitivity. The hydrogel was prepared through a one-step route under low temperature, with long wavelength-emissioned G-CDs as fluorescence probe and NIPAM as monomers. The optical characterization experiments show that the prepared hydrogel exhibits classic excitation wavelength-dependent photoluminescence properties. The experiments also show that the hydrogel exhibits sensitive response to temperature with excellent reversibility and repeatability, and it also exhibits certain pH-response property and excellent biocompatibility. The prepared CDs/PNIPAM hydrogel can be used as fluorescent ink, and it emits clear and bright fluorescence under UV radiation. This composite hydrogel provides new fluorescence/temperature-sensitive material for the fields of portable temperature-sensitive sensors, drug carrier, cell imaging, fluorescent ink and anti-counterfeit identification.

## Figures and Tables

**Table 1:** Zeta potentials of G-CDs, PNIPAM, and CDs/PNIPAM.

Samples	Zeta Potential (mV)
G-CDs	+11.4
PNIPAM	−14.4
CDs/PNIPAM	−3.36

## Supplementary Materials

The following are available online at https://www.mdpi.com/2073-4360/11/7/1171/s1.
