# Supplementary material for: The Preparation of Green Fluorescence-Emissioned Carbon Dots/Poly(N-Isopropylacrylamide) Temperature-Sensitive Hydrogels and Research on Their Properties
Source: Polymers (Basel). 2019 Jul 11;11(7):1171. doi: 10.3390/polym11071171 (PMC6680904; doi:10.3390/polym11071171)
Supplement: Supplementary file 1 [file polymers-11-01171-s001.pdf]

Supplementary Materials

The Preparation of Green Fluorescence-Emissioned Carbon Dots/Poly(*N*-isopropylacrylamide) Temperature-Sensitive Hydrogels and Research on Their Properties

Dan Zhao[1,2], Wenting Ma[1,2], Rong Wang [1], Xinzhou Yang [1,2], Jun Li [1,2], Ting Qiu [3], Xincai Xiao [1,2,*]

[1] School of Pharmaceutical Sciences, South-Central University for Nationalities, Wuhan 430074, P. R. China

[2] National Demonstration Center for Experimental Ethnopharmacology Education (South-Central University for Nationalities), Wuhan 430074, P. R. China

[3] Information Center, Shanghai Institude of Organic Chemistry, Shanghai 200032, P. R. China

[*] Correspondence: xcxiao@mail.scuec.edu.cn (X.C.X.); Tel.: +86-189-7118-1538 (X.C.X.)

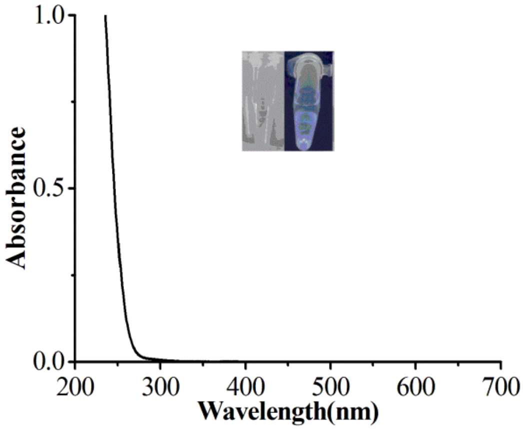

Figure S1. UV-Vis spectrum of PNIPAM. Illustration: left is daylight; right is ultraviolet.

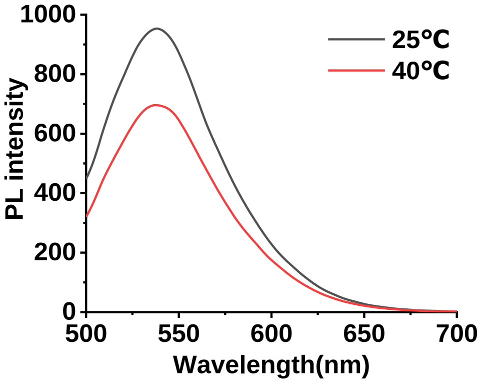

Figure S2. Fluorescence spectra of CDs/PNIPAM at 25 and 40 ℃.

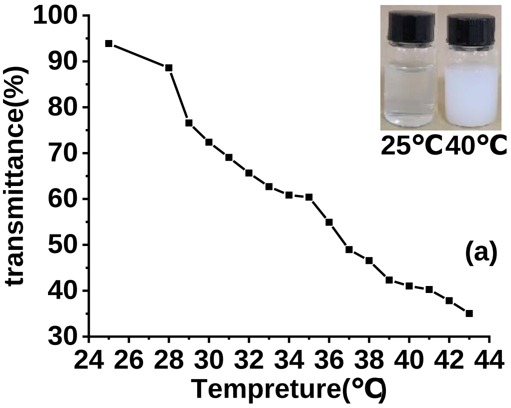

Figure S3. Change of PNIPAM particle transmittance at (a) different temperatures. (Inset: photos of PNIPAM particle at different temperatures.)

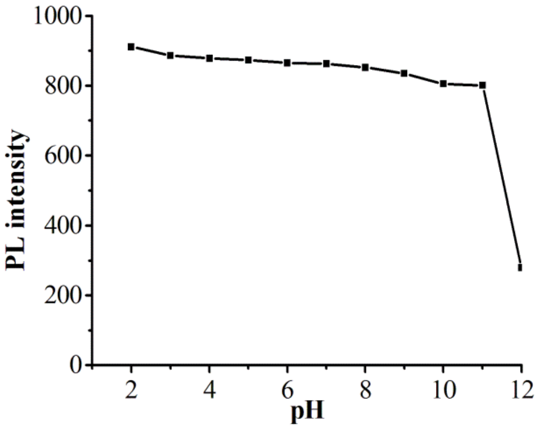

Figure S4. Fluorescence intensity of G-CDs at different pH conditions.

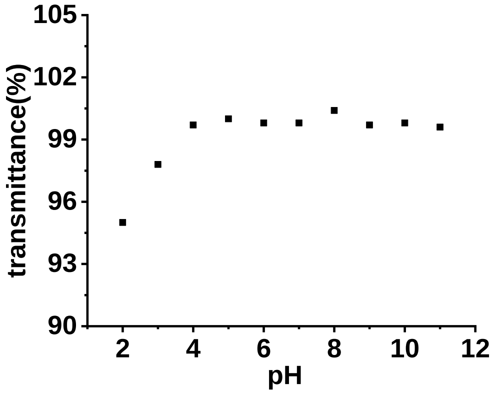

Figure S5. Change of PNIPAM particle transmittance at different pH buffer solvents.
